# Anatomical and Physiological Plasticity in *Leymus chinensis* (Poaceae) along Large-Scale Longitudinal Gradient in Northeast China

**DOI:** 10.1371/journal.pone.0026209

**Published:** 2011-11-08

**Authors:** Renzhong Wang, Wenwen Huang, Liang Chen, Linna Ma, Chengyuan Guo, Xiaoqiang Liu

**Affiliations:** 1 State Key Laboratory of Vegetation and Environmental Change, Institute of Botany, Chinese Academy of Sciences, Beijing, China; 2 College of Life Sciences, Hubei Normal University, Hubei, China; 3 Department of Earth Sciences, Indiana University-Purdue University Indianapolis, Indianapolis, Indiana, United States of America; University of California, United States of America

## Abstract

**Background:**

Although it has been widely accepted that global changes will pose the most important constrains to plant survival and distribution, our knowledge of the adaptive mechanism for plant with large-scale environmental changes (e.g. drought and high temperature) remains limited.

**Methodology/Principal findings:**

An experiment was conducted to examine anatomical and physiological plasticity in *Leymus chinensis* along a large-scale geographical gradient from 115° to 124°E in northeast China. Ten sites selected for plant sampling at the gradient have approximately theoretical radiation, but differ in precipitation and elevation. The significantly increasing in leaf thickness, leaf mass per area, vessel and vascular diameters, and decreasing in stoma density and stoma index exhibited more obvious xerophil-liked traits for the species from the moist meadow grassland sites in contrast to that from the dry steppe and desert sites. Significant increase in proline and soluble sugar accumulation, K^+^/Na^+^ for the species with the increasing of stresses along the gradient showed that osmotic adjustment was enhanced.

**Conclusion/Significance:**

Obvious xerophytic anatomical traits and stronger osmotic adjustment in stress conditions suggested that the plants have much more anatomical and physiological flexibilities than those in non-stress habitats along the large-scale gradient.

## Introduction

Most climate change scenarios have suggested that the increase in drought and high temperature pose the most important environmental constrains to plant survival, productivity and vegetation dynamics [1], [2], [3], [4], thus studies of plant responses to these environmental changes as well as its adaptive strategies are becoming increasingly important. Many studies on plant response and adaptability to environment changes were developed over the past couple of decades, including population quantity [5], morphological traits [6], [7], anatomical structures and physiological processes [6], [8], as means of studying plant responses to environment changes, such as changing in geographical range, rainfall, latitude and altitude. Few examined the influences of large-scale climate variability on tree mortality [4] and grass population and resource partitioning [9]. But anatomical and physiological plasticity of plants to large-scale natural environmental gradient and their subsequent ecological implications with global climate changes have remain largely unexplored. Understanding of plant response and adaptability to natural environments with difference in temperature and precipitation along large-scale spatial gradients may help us to understand how plant will respond and adapt to temporal climate change [5], [8], which is also important to predict the dynamics of natural vegetation with global changes.

Plant responses to environmental changes (*e.g.* precipitation and temperature) are complex, and can be divided into several strategies (*e.g.* plasticity and tolerance), even though they are not mutually exclusive and plants may combine a range of rezones types [2], [8]. In generally, the common mechanism for plant successfully to tolerate drought and high temperature is associated with a variety of adaptive strategies, involving minimizing water loss and maximizing water uptake. Water loss is minimized by reducing light absorbance through rolled leaves, increasing leaf mass per area (LMA), thickening leaf blade and closing stomata [10], [11]. Water uptake is maximized by increasing investment in roots and vessel number, reducing vessels size in stems [12], [13]. Moreover, tolerance to low tissue water potential may involve accumulation of proline and soluble sugar contents and K^+^/Na^+^ selectivity, for their high accumulation in cells is associated with the prevention of protein denaturation under the stress, maintenance of osmotic adjustment and cell turgor [14]. These strategies for plant successfully to tolerate environmental changes are well documented and widespread. However, most previous studies related to these strategies were based on short-term growth experiments (less than 4 months) at controlled conditions and small-scales or on species with relative simple life histories [15], the adaptive strategies in perennial natural plants with long-term growth at large-scale geographical gradient remains unclear.

*Leymus chinensis* (Trin.) Tzvel., a perennial rhizomatous C_3_ cosmopolitan grass, is widely distributed at eastern Eurasian steppe zone, from moist regions in western part of the Northeast Plain to dry regions in the eastern part of the Mongolian Plateau, China [16]. Xerophytic traits, *e.g.* thick rhizome systems, high plasticity in leaf thickness and LMA, enable the species to successfully tolerate drought when soil moisture is less than 4% in dry seasons in desert grasslands [8]. Widely distribution of the cosmopolitan grass makes it an ideal plant species for studying plant plasticity to large-scale environmental changes. Studies on the species and its response to large-scale climatic variables have been conducted in variations of population density, plant height, leaf size, biomass and biomass allocation [5], [17]. However, the anatomical and physiological plasticity in the species to **l**arge-scale environmental changes have not yet been examined. In this study, we first hypothesize that the adaptability of *L. chinensis* was strongly enhanced by the obvious xerophytic anatomical traits and relative greater osmotic adjustment with the increase of drought along the large-scale gradient. We further hypothesize that the decrease of precipitation from the east to the west along the gradient was the critical factor related with adaptive strategies of *L. chinensis*. Our research aims to better understand how plant responses to large-scale environmental changes in terms of their adaptive strategies to stresses with global changes.

## Results

### Leaf thickness and LMA

**Figure 1:**
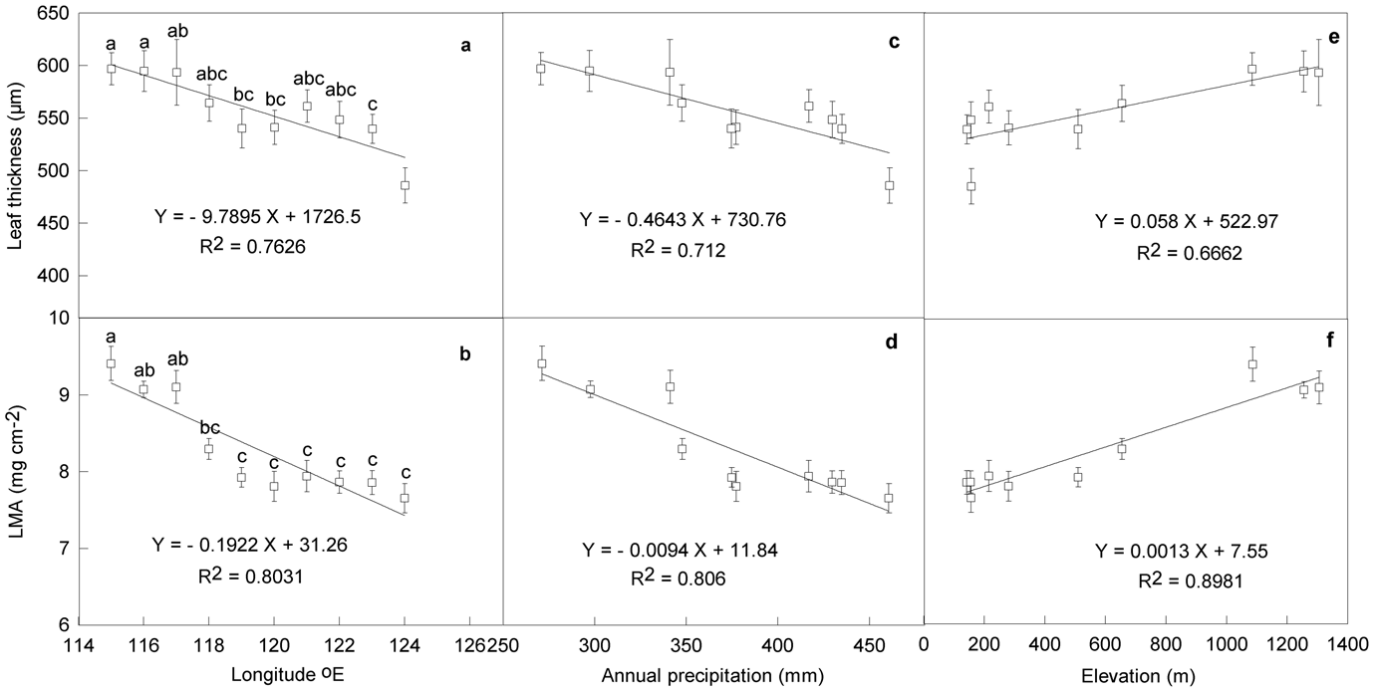
Variations of leaf thickness (a) and leaf mass per unit area (LMA) (b) in *Leymus chinensis* along large-scale longitudinal gradient and their correlations with annual precipitation (c, d) and elevation (e, f) at the gradient in northeast China. Bars are means (± SE) of 25–30 replications. Bars with the same lowercase letters indicate no statistically significant differences (P > 0.05).

Leaf thickness of *L. chinensis* exhibited remarkable variations along the large-scale gradient due to the variations of precipitation (Fig. 1a). The leaf thickness of the species at the west end (115°E) was about 22.8% higher than that at the east end (124°E) (P<0.001). Leaf thickness showed no significant differences among the neighbor sites, *e.g*. sites from 115° to 117°E and from 122° to 123°E (P > 0.05). Average leaf thickness of the sites from meadow grasslands (from120° to 124°E) was about 8% lower than that from typical steppes and desert grasslands (from 115° to 119°E) (P<0.01). Leaf mass per area (LMA) in the species varied considerable along the gradient (Fig. 1b). On average, typical steppe and desert grassland sites had high LMA (8.76 mg/cm^2^), while that from meadow grassland sites had low LMA (7.82 mg/cm^2^), and the differences were significant (P<0.05). The difference between neighbor sites was not significant (P > 0.05), but the differences were magnified for the sites at the two ends of the gradient. Leaf thickness and LMA were strongly and negatively correlated with annual precipitation (R^2^ = 0.712, P<0.01; R^2^ = 0.806, P<0.01) (Fig. 1c, d), but positively correlated with elevation (R^2^ = 0.6662, P<0.01; R^2^ = 0.8981, P<0.001), respectively along the gradient (Fig. 1e, f).

### Epidermal cell, stoma density and stoma index

Epidermal cell densities in *L. chinensis* varied slightly along the large-scale gradient (Table 1), with very few significant effects (P<0.05). Of the 10 sites, the epidermal cell densities in Molimiao (121°E) and Linxi (118°E) sites were significantly lower than those in Changling (123°E) and Jiamatu (122°E) sites (P<0.05). Those at the other sites, however, did not differ remarkably (P > 0.05), even though the average epidermal cell density from meadow grassland sites was about 5% higher than that from typical steppe and desert grassland sites (P > 0.05).

**Table 1:** Anatomical variations in *Leymus chinensis* at large scale longitudinal gradient in northeast China.

Sites	Epiderm cell density (mm^2^)	Stoma density (mm^2^)	Stoma index	Vessel diameter (µm)	Vascular diameter (µm)	Vascular distance (µm)	Vd/Lt
124°E	330.4±5.4^a b^	125.0±4.7	0.378±0.011	52.01±1.27	226.78±5.60	799.71±35.52^a^	0.461±0.018^a^
123°E	345.9±3.6^b^	114.9±3.9	0.332±0.011^a^	54.84±1.74	237.31±4.82^a^	800.83±19.14^a^	0.444±0.012^a^
122°E	341.3±3.7^b^	111.5±5.8	0.327±0.017^a^	58.08±1.38^a^	240.36±7.12^b^	832.62±64.43	0.444±0.020^a^
121°E	305.1±3.2^a^	101.6±4.3^a^	0.331±0.014^a^	66.91±1.02^b^	241.94±9.08^b^	865.49±27.77	0.426±0.020^a^
120°E	338.3±11.1^b^	100.9±3.0^a^	0.301±0.014^b^	61.46±1.43^b^	234.72±3.56^a^	891.27±29.07^b^	0.445±0.014^a^
119°E	313.5±13.9^a b^	93.5±6.2^b^	0.297±0.009^b c^	63.61±1.66^b^	239.81±6.56^b^	895.89±37.45^b^	0.447±0.008^a^
118°E	301.0±9.7^a^	102.3±6.3^a^	0.338±0.011^a^	59.34±1.34^a b^	246.87±4.34^c^	962.55±24.47^c^	0.442±0.010^a^
117°E	314.1±10.7^a b^	100.5±4.9^a^	0.320±0.009^a^	70.34±1.40^c^	245.55±8.68^c^	920.07±45.28^d^	0.423±0.012^a^
116°E	318.0±11.9^a b^	89.2±6.3^b^	0.279±0.013^c^	74.52±1.09^c^	256.70±7.23^d^	978.67±50.01^c^	0.429±0.009^a^
115°E	334.6±8.0^a b^	90.9±3.7^b^	0.271±0.008^c^	68.28±1.67^c^	251.36±5.10^d^	941.43±37.64^d^	0.426±0.010^a^

Values are means (± SE) of 25–30 replications. Values with the same letters indicate no significant difference between sites (site/site) within each anatomical property (P > 0.05).

Stoma densities and stoma index, however, differed significantly along the large-scale gradient (Table 1). Stoma density in the species decreased from 125.0 /mm^2^ at the east end (124°E site) to 90.9/mm^2^ at the west end (115°E site); the average stoma density from the meadow grassland sites was about 16.25% greater than that from typical steppe and desert sites (P<0.05). Stoma index exhibited a similar pattern of stoma densities along the gradient, decreasing from 0.378 at the east end to 0.271 at the west end. Both stoma density and stoma index in the species were positively correlated with annual precipitation (R^2^ = 0.8359, P<0.01; R^2^ = 0.6824, P<0.01) and precipitation in growing season (R^2^ = 0.8728, P<0.001; R^2^ = 0.7280, P<0.01), respectively. Stoma density was also correlated with elevation (R^2^ = 0.6598, P<0.05) along the gradient.

### Vessel and vascular diameters

Unlike stoma density and stoma index, vessel and vascular diameters in the species increased from the moist east end (124°E site) to the dry west end (115°E site) of the gradient (Table 1). The average vessel diameters and vascular diameters at typical steppe and desert grassland sites were 14.6% (P<0.01) and 5.0% (P<0.05) greater than those from the meadow grassland sites, respectively. But those between the neighbor sites did not differ significantly (P > 0.05). Vessel diameters of the species were negatively and significantly correlated with annual precipitation (R^2^ = 0.6459, P<0.05) and precipitation in growing season (R^2^ = 0.8431, P<0.01), respectively, but positively correlated with elevation (R^2^ = 0.6652, P<0.01) along the large-scale gradient.

Although vascular distances of the species varied little along the large-scale gradient (Table 1), it also increased from the moist east end to the dry west end. The average vascular distances from the west end (115–116°E) were about 20% higher than those from the east end (123–124°E) (P<0.001). On the contrary, Vd/Lt for the species decreased from the east to the west of the gradient (Table 1) with the Vd/Lt at 124°E site about 8% greater than that at 115°E site, even though the differences among the 10 sites were not significant (P > 0.05).

### Proline and soluble sugar contents

**Figure 2:**
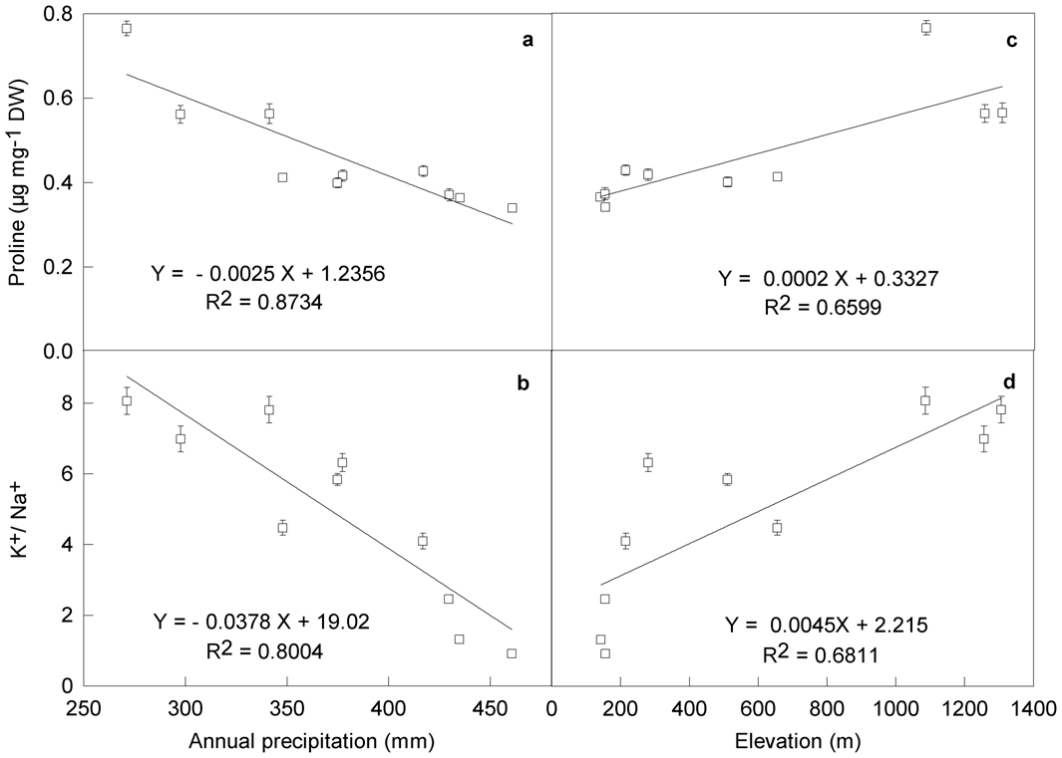
Correlations of proline content and K^+^/Na^+^ in *Leymus chinensis* with annual precipitation (a, b) and elevation (c, d) along large-scale longitudinal gradient in northeast China. Bars are means (± SE) of 30–35 replications.

Both proline and soluble sugar contents in *L. chinensis* changed significantly with climatic and geographic variables along the gradient (Table 2). Proline content in the species increased from the east end to the west end. Average proline content was 0.340 µg mg^−1^DW at Chaganhua site (124°E), which was about 1/2 of that at Abagaqi site (115°E) (P<0.01). Soluble sugar content exhibited a similar pattern of proline along the large-scale gradient. The average proline and soluble sugar contents from the dry sites (115–119°E) were about 40.8% and 63.1% greater (P<0.01) than those from the moist sites (124–120°E), respectively. Proline contents were negatively and significantly correlated with annual precipitation (R^2^ = 0.8734, P<0.001) (Fig. 2a) and annual air temperature (R^2^ = 0.8619, P<0.001), but positively correlated with elevation (R^2^ 0.6599, P<0.05) (Fig. 2c).

**Table 2:** Proline, soluble sugar, [K^+^], [Na^+^] and K^+^/ Na^+^in *Leymus chinensis* at large scale longitudinal gradient in northeast China.

Sites	Proline(µg/mgDW)	Soluble sugar(µg/mgDW)	[K^+^](µmol/gDW)	[Na^+^](µmol/gDW)	K^+^/ Na^+^
124°E	0.340±0.007^a^	5.64±0.272^a^	150.61±1.73^a^	165.62±3.53^a^	0.913±0.023
123°E	0.364±0.009^a b^	5.57±0.428^a^	215.80±3.44^b^	166.52±5.81^a^	1.314±0.056
122°E	0.371±0.014^a b c^	5.49±0.148^a^	267.20±7.46^c^	109.11±2.31	2.460±0.089
121°E	0 427±0.013^c^	10.59±0.312^b^	266.57±7.29^c^	65.15±1.73	4.102±0.224^a^
120°E	0.417±0.014^b c^	10.26±0.127^b^	289.75±7.00^c^	45.54±0.69^b^	6.319±0.235^b c d e^
119°E	0.399±0.011^b c^	10.99±0.289^b^	231.69±7.09^b c^	40.39±0.79^c^	5.838±0.167^b d^
118°E	0.412±0.009^b c^	12.68±0.298^c^	189.08±7.56^b^	41.97±1.24^b c^	4.475±0.213^a^
117°E	0.563±0.024^e^	12.70±0.395^c^	208.76±3.32^b^	27.33±1.27^d^	7.814±0.375^c d e^
116°E	0.562±0.021^e^	12.28±0.473^b c^	184.29±9.03^a b^	25.99±0.57^d^	6.988±0.368^d e^
115°E	0.765±0.017	12.58±0.276^c^	206.85±6.45^b^	25.42±0.94^d^	8.067±0.382^e^

Values are means (± SE) of 30–35 replications. Values with the same letters indicate no significant difference between sites (site/site) within each physiological property (P>0.05).

### K^+^ and Na^+^contents

Variation of [K^+^] in the species showed patterns different from those of proline and soluble sugar contents, first increasing, maximum at 120°E and then decreasing with increasing drought along the large-scale gradient (Table 2). There was no significant difference in [K^+^] of the neighbor sites, *e.g.* 122–119°E sites and 119–115°E sites (P > 0.05). [Na^+^] of the species, however, decreased significantly from the east to the west of the gradient, with very remarkable effects (P<0.05). The [Na^+^] at the 115°E site was only 1/6 of that at the 124°E site (P<0.001).

Unlike [Na^+^], K^+^/ Na^+^ for the species varied significantly along the large-scale gradient and was significantly correlated with climatic and geographic variables. K^+^/Na^+^ in the dry 115°E sites was about 9 times of that in the moist 124°E sites (P<0.001), but the differences in K^+^/Na^+^ between neighbor sites were much less at the middle (121–118°E sites) and the west (117–115°E sites) parts of the gradient (P > 0.05). Along the gradient, K^+^/Na^+^ of the species were negatively and strongly correlated with annual precipitation (R^2^ = 0.8004, P<0.001) (Fig. 2b), but positively correlated with elevation (R^2^ = 0.6811, P<0.01) (Fig. 2d).

## Discussion

Large-scale climatic and geographic changes, due to natural and human activities, are known to affect plant survivals and distributions directly through influences on plant responses and adaptive strategies [2], [17], [18], [19]. But, most studies on plant responses and adaptive strategies over the past couple of decades, covering subjects from plant plasticity to physiological and biochemical processes in controlled stresses [11], [12], [20], focused on either responses or plasticity to the controlled stress status based on short-term growth experiments, shed little light on anatomical and physiological plasticity of mature plants with long-term growth along large-scale gradients in natural ecosystems. Moreover, some large-scale studies have also demonstrated that broad-scale environmental variations can influence physiological traits in ways that have previously not been recognized [8], [19]. This knowledge on anatomical and physiological plasticity for mature plants is essential for our ability to predict how plants will respond and adapt to temporal variables with global changes and to explore the fate of natural ecosystems in long-term climate change.

Along the large-scale gradient in northeast China, drought is the major environmental factor affecting plant survival, distribution, resource partitioning, growth and reproduction for *L. chinensis* [5], [18]. The anatomical and physiological changes in *L. chinensis* suggested that the species has multiple strategies to tolerate water scarcity under the broad-scale environmental variations. In present study, from the moist meadow grassland sites to the dry steppe and desert sites, the significant increase in leaf thickness and LMA exhibited more obvious xerophil-liked traits (Fig. 1). Higher LMA was proved to be a main adaptive strategy to droughts for higher LMA, which was positively related with photosynthetic tissue per area and investment in structural tissues, contributing to higher tolerance in unfavorable conditions [8], [21]. Higher LMA for the species with the increase of drought along the gradient mainly resulted from the greater leaf thickness because there was no significant difference in leaf area in the species among the sites [9]. Moreover, relative higher LMA and leaf thickness at dry regions may be evolutionarily favored for efficient water condition, because high LMA and leaf thickness enable plants to maintain relative high leaf water content at the dry stresses. Some studies also demonstrated that leaf thickness was correlated negatively with the hydraulic resistance of plant leaf [22], [23], [24]. These explanations are supported by the strong negative correlations between this two leaf traits and annual precipitations (Fig. 1c, d). The positive correlations of both leaf thickness and LMA with elevation (Fig. 1e, f) suggested that the plasticity of leaf traits is an important adaptive strategy for the species to tolerate broad-scale environmental variations in the region.

With the increase of drought along the large-scale gradient, both stoma density and stoma index in *L. chinensis* decreased significantly from moist meadow grassland sites to dry steppe and desert sites (Table 1), and average stoma density and stoma index at the dry sites decreased 14.0% and 9.9% compared with those at the moist sites. Relative lower stoma density and stoma index for the species enables it to minimize water loss by leaf transpiration and increase water-use efficiency, and this is in consistent with observations in drought and salt stress [25], [26]. The decrease of stoma index was mainly due to the decline of stoma density, because there was slightly variation in leaf epidermal cell densities in *L. chinensis* along the gradient (Table 1). The strong positive correlations of stoma density and stoma index with annual precipitations also supported that anatomical plasticity is an important strategy for the species to adapt large-scale water scarcity.

Unlike the studies at salt stresses [8], [13], the species at the drier sites had relative larger vessel and vascular diameters, and vascular distances (Table 2). There have been debates on vessel actions in plant water conductance, for example, whether there is a negative relationship between vessel diameter and water flow. In generally, high salinity may result in narrow vessels and increase vessel density, which can maximise water uptake in salt stress [13]. In some studies, embolism and cavitation, which reduce the ability of xylem water conductance, increase linearly with vessel diameter in fern and wood plants [27], [28]. On the contrary, the observations by Sobrado [13] indicated that a higher density of narrow vessels would not compensate for large vessels in term of potential for water flow, and large vessels allow for low investment in xylem structures while maintaining high permeability. This explanation is supported by the other observations found in *L. chinensis*, vessel, vascular and xylem diameters being larger at drier condition [8]. Moreover, in some cases, leaves had been found to be less vulnerable to embolism than stems [23], [24], leaf xylem conduit trend to maximize hydraulic conductance, which was inconsistent with that in stem. With the increase of drought along the large-scale gradient, the increases in vessel and vascular diameters are favorable strategies for the grass species to tolerate drought, because relative larger vessel and vascular diameters, and vascular distances enable plants to maximize hydraulic conductance and to miximize investment in xylem structures. This was supported by the strong negative correlations between vessel diameters and annual precipitations along the gradient. It was also supported indirectly by the decreasing of Vd/Lt at the gradient (Table 1), which indicated leaf soft tissue proportion increased with drought and the plants had greater capacity to maintain relative high leaf water content at the dry conditions. Small Vd/Lt allows for low investment in xylem structures for the grass species at dry steppe and desert sites. The findings (*e.g.* large plasticity in leaf thickness and LMA, vessel and vascular diameters, stoma density and stoma index) supported our hypothesis that the adaptability of *L. chinensis* was strongly enhanced by the obvious xerophytic anatomical traits along the large-scale gradient.

Physiological plasticity of *L. chinensis* exhibited its higher tolerance to drought stress. The increase of proline and soluble sugar contents in the species indicated the capacity for dehydration tolerance and the osmotic adjustment were enhanced with the increase of drought along the large-scale gradient (Table 2). Osmotic adjustment is considered one of the crucial processes for plant to adapt water deficit for cell osmotic potential decreases in water deficit conditions allowing the maintenance of water absorption and cell turgor in drought [2], [8]. Many studies suggested that accumulation of free proline and soluble sugar in plant leaves is one of the most common and direct biochemical adaptive strategies to water deficit, because there are positive correlations between the capacity for proline and soluble sugar accumulation and dehydration tolerance [29], [30], [31]. The significant and negative correlations between proline contents and annual precipitations and annual air temperature, and the positive correlations between proline contents and elevation along the gradient (Fig. 2) also suggests that proline and soluble sugar accumulations are the favorable strategies for the species to adapt dry stress. Observations by Chen and Wang [8] indicated that there was a positive correlation between relative water content and proline and soluble sugar accumulation in *L. chinensis* in drought, this demonstrated that free proline and soluble sugar accumulation had taken part in osmotic adjustment and enabled cell to maintain turgor during drought and saline stresses [8].

Lower [Na^+^] and high K^+^/Na^+^ for the species at dry sites along the gradient showed its higher tolerance to drought stresses (Table 2). Lower Na^+^ and high K^+^/Na^+^ in the species at drought enable plants to avoid ion toxicity and maintain photosynthesis. Salt in soil solution reduces plant capacity to take up water and lower the external water potential in long-term growth of mature natural plants, leading to slower growth. Excessive amounts of salt accumulate in leaves and reach toxic levels, causing necrosis and reducing the photosynthetic area, and subsequently further decline in plant growth [32], and the effect is more strong in dry condition [8]. The negative correlations between K^+^/Na^+^ and precipitations indicated that K^+^/Na^+^ is one of the critical strategies to affect plant survivals, distributions for *L. chinensis* along the large-scale gradient (Fig. 2). The significant variations in proline and soluble sugar accumulation, K^+^/Na^+^ for the species with the increase of stresses (including drought, temperature and elevation) from the east to the west of the large-scale gradient imply that plants in stress conditions should show much more physiological flexibility than those in un-stress habitats.

Plant strategies are combined with a mixture of response types to tolerate stresses in practice [2]. The findings of this study showed that obvious xerophytic anatomical traits and stronger osmotic adjustment of *L. chinensis* with the increase of stresses along the gradient are combination of adaptive strategies to survive the broad-scale environmental variations in Northeastern China. There were significant correlations among the anatomical and physiological traits (Table 3), as well as between the anatomical plasticity and osmotic adjustment (*e.g.* proline and soluble sugar accumulation and K^+^/Na^+^) (P<0.05). This combination of mixture strategy types promotes plants to tolerate large-scale environmental stresses (*e.g.* drought and temperature changes) successfully, because the function of anatomical and physiological strategies is compensatory [8]. The significant relationships between both anatomical and physiological plasticity and precipitation support our hypothesis that the decrease of precipitation from the east to the west along the large-scale geographical gradient was one of the critical factors related with adaptive strategies of *L. chinensis* in the study area. These results suggested that the plants in environmental stresses (*e.g.* drought and salt) should have much more anatomical and physiological plasticity than those in non-stress conditions, this may explain the fact that *L. chinensis* can widely distribute at eastern Eurasian steppe zone, from moist meadows in western part of the Northeast Plain to dry typical steppes and desert grasslands in the Mongolian Plateau.

**Table 3:** Correlations between anatomical and physiological traits (trait/trait) in *Leymus chinensis* at large scale gradient in northeast China (* P<0.05, ** P<0.01).

	Leaf thickness	LMA	Stoma density	Stoma index	Vessel diameter	Vascular diameter	Vascular distance	Proline	Soluble sugar	[Na^+^]	[K^+^]
LMA	0.870**										
Stoma density	−0.801**	−0.672*									
Stoma index	−0.737*	−0.646*	0.894**								
Vessel diameter	0.863*	0.785**	−0.869**	−0.748*							
Vascular diameter	0.930**	0.837**	−0.808**	−0.712*	0.823**						
Vascular distance	0.781**	0.746*	−0.856**	−0.652*	0.770**	0.839**					
Proline	0.791**	0.927**	−0.691*	−0.734*	0.730*	0.725*	0.651*				
Soluble sugar	0.752*	0.708*	−0.856**	−0.606	0.797**	0.718*	0.925**	0.665*			
[Na^+^]	−0.772**	−0.660*	0.921**	0.723*	−0.835**	−0.723*	−0.908**	−0.647*	−0.941**		
[K^+^]	0.077	−0.319	−0.183	−0.275	0.071	−0.114	−0.140	−0.149	−0.051	−0.181	
K^+^/Na^+^	0.793**	0.783**	−0.893**	−0.810**	0.860**	0.689*	0.834**	0.797**	0.888**	−0.936**	0.113

## Materials and Methods

### Study sites

The experiment was conducted on a large-scale geographical gradient, ranging from 43°16′ to 44°35′ N; 115°43′ to 124°16′ E, about 900 km from the west to the east, from 2008 to 2009 (Table 4). 10 sites, selected for plant sampling on the gradient, have similar light regimes, but differ in longitude, precipitation and altitude. Native grasslands dominated by *L. chinensis* are widely distributed on the large-scale gradient. Due to the steep decrease of precipitation from the east to the west, vegetations vary gradually from moist meadows in the east to typical steppes and desert grasslands in the west with agricultural fields and shrubs in the middle [5], [18]. *L. chinensis* meadow grasslands along the gradient have dark meadow soil and chernozem in the east, while those of typical steppes and desert grasslands have chernozem and chestnut in the west. The elevations of the gradient increased from 142–156 m above sea level (a.s.l.) in the east to 1086–1306 m a.s.l. in the west (Table 4). For at least 10 years prior to 2009, the sites, selected for the study, had never been grazed, ploughed, fertilized or burned, but transient floods may occur in the eastern meadows.

**Table 4:** Sample site locations and properties at the large scale gradient in northeast China.

Sites	Locations	Elevation(m)	Vegetation type	Soil type	Annual prec.(mm)	Annual temp.(°C )	Aridity index
Chaganhua	44°35′N 124°16′ °E	156	Meadow	Dark meadow	460.5	4.9	1.6
Changlin	44°34′N 123°31′ °E	142	Meadow	Dark meadow	434.7	4.8	1.7
Jiamatu	44°12′N 122°49′ °E	155	Meadow	Dark meadow	429.5	5.2	1.8
Molimiao	43°35′N 121°51′ °E	215	Meadow	Chernozem	416.8	5.7	2.2
Shaogen	43°38′N 120°47′ °E	280	Meadow	Chernozem	377.2	5.2	2.1
Balin	43°57′N 119°20′ °E	510	Steppe	Chernozem	374.7	4.8	2.1
Linxi	43°36′N 118°22′ °E	655	Steppe	Chernozem	347.8	4.9	2.1
Keshiketengqi	43°16′N 117°09′ °E	1306	Steppe	Chestnut	341.2	3.2	2.2
Xilinguole	43°32′N 116°40′ °E	1256	Steppe	Chestnut	297.6	1.7	2.2
Abagaqi	43°55′N 115°43′ °E	1086	Desert grassland	Chestnut	271.2	1.1	2.4

(*Abbreviations* - prec., precipitation; temp., temperature).

### Climate

The study area has a continental monsoon climate, with large seasonal temperature and precipitation variations. Moisture along the large-scale gradient varies steeply, with annual precipitation ranging from 460 mm at the east end to 270 mm at the west end (Table 4). Precipitation is not distributed evenly over growing season, of which 70% falls between June and August. The mean annual air temperature in the area ranges from 6°C to 1°C, varying from - 21°C in January to 23°C in July. A more detailed description of the climate in the region and the correlation coefficients among longitude, elevation and climatic factors can be found in Zhang *et al.* [18] and Ni & Zhang [33].

### Methods

About 1–2 ha typical native *L. chinensis* grassland was selected for plant sampling at each site. 3–4 sample plots (20×20 m) with uniform soil and even distribution of the species were established at each site and plants in each plot were sampled using 4–5 randomly located 1×1 m quadrats. All the quadrats at each site have similar light regimes and precipitation. Samplings were carried out in June and July.

### LMA measurement

Within each site, 2nd fully expanded leaves (2nd leaf from shoot top, hereafter) were sampled randomly from 3–5 mature plants in each quadrat at about 9:00 am. Leaf area was measured using a flatbed scanner connected to a personal computer running image analysis software. All leaf samples were placed in perforated paper bags separately and oven-dried at 80°C for 24 h, then weighed to measure dry leaf mass. Leaf mass per area (LMA) was expressed as leaf dry mass per area.

### Leaf anatomy

5–6 sections (2 cm) were cut from the middle of 2nd leaves of mature plants in each quadrat and fixed in FAA (3.7% formalin, 50% ethanol and 5% acetic acid). Leaf slides of 8–10 µm were obtained with a rotary microtome (Leitz, Wetzlar, Germany), leaf thickness, diameters of vessels and vascular bundles, and vascular distances were measured. A more detailed description of leaf anatomy can be found in Chen & Wang [8] and Sobrado [13].

Leaf pieces (1×1 cm) were treated with bleach (NaOCl) to eliminate the mesophyllic tissue. Once the two epidermises were separated and the leaf mesophyllic remains were eliminated, the pieces were stained with safranin and mounted in glycerol. From these leaf preparations, epidermal cells and abaxial stomata were counted for leaf samples [8], [13].

### Proline and soluble sugar contents, [K^+^] and [Na^+^]

For praline and soluble sugar contents, [K^+^] and [Na^+^] analysis, 10 g 2nd leaf samples of the species were taken from each quadrat, respectively, with 30–35 replications in each site. The samples were oven-dried at 80°C for 24 h to constant weight and ground using a mortar and pestle to pass though a 100- mesh screen.

0.5 g powder samples were added with sulphosalicylic acid (10 ml, 3%), and the extract was filtered through filter paper. 2 ml aliquots were taken for proline estimation by the acid-ninhydrin method [8], [34].

Approximately 50 mg leaf powders of each sample were extracted with 80% ethanol (v/v) at 85°C for 1 h. The solutions were then centrifuged at 12 000 *g* for 10 min. Ethanol extraction step was repeated three times and the three resulting supernatants were combined, treated with activated charcoal, and evaporated to dryness in a vacuum evaporator. Residues were redissolved in distilled water, and subjected to soluble sugar analysis using the anthrone-sulfuric acid method [35].

Approximately 50 mg leaf powders were extracted in 10 ml hot water (70°C for 3 h) and the solutions were then centrifuged at 12 000 *g* for 10 min. The concentrations of K^+^ and Na^+^ in dilutions were determined with flame photometry (Flame Photometer 410, Corning Halstead, UK) [8].

### Statistical analysis

All statistical analyses were performed using SPSS 13.0 (SPSS for Windows, Chicago, IL, USA). Stoma index refers to the ratio of stoma number to the total number of epidermal cells. Vd/Lt, an anatomical indicator of soft tissue proportion, was calculated by vascular diameter/ leaf thickness [8]. Plant variables in each vegetation type, e.g. LMA, stoma density and proline of meadow grassland, typical steppe, desert grassland, refer to the average value of each plant trait (e.g. LMA, stoma density and praline) from the sites in each vegetation type. Difference in each parameter between sites was tested using one-way analysis of variance (ANOVA) (P<0.05). Regression of plant parameters, *e.g.* leaf thickness, LMA, proline, soluble sugar and K^+^ /Na^+^ against longitude, elevation, and climate parameters, *e.g.* annual precipitation, annual air temperature, and mean month temperature were performed using SigmaPlot 10.0 in order to explain the spatial variations of anatomical and physiological plasticity of *L. chinensis* along the large-scale gradient. Climate data (1980 to 2010) were taken from the Climate Database of State Key Laboratory of Vegetation and Environmental Change, Institute of Botany, and some from local weather stations throughout the large-scale gradient.

## References

[1] Castro-Díez P, Villar-Salvador P, Pérez-Rontomé C, Maestro-Martínez M, Montserrat-Martí G (1997). Leaf morphology and leaf chemical composition in three *Quercus* (Fagaceae) species along a rainfall gradient in NE Spain.. Trees-Structure and Function.

[2] Chaves M, Pereira J, Maroco J (2003). Understanding plant responses to drought - from genes to the whole plant.. Functional Plant Biology.

[3] Field R, O'brien E, Whittaker R (2005). Global models for predicting woody plant richness from climate: development and evaluation.. Ecology.

[4] Villalba R, Veblen T (1998). Influences of large-scale climatic variability on episodic tree mortality in northern Patagonia.. Ecology.

[5] Wang RZ, Gao Q (2003). Climate-driven changes in shoot density and shoot biomass in *Leymus chinensis* (Poaceae) on the North-east China Transect (NECT).. Global Ecology and Biogeography.

[6] Cordell S, Goldstein G, Mueller-Dombois D, Webb D, Vitousek P (1998). Physiological and morphological variation in Metrosideros polymorpha, a dominant Hawaiian tree species, along an altitudinal gradient: the role of phenotypic plasticity.. Oecologia.

[7] Li B, Suzuki J, Hara T (1998). Latitudinal variation in plant size and relative growth rate in *Arabidopsis thaliana*.. Oecologia.

[8] Chen L, Wang RZ (2009). Anatomical and physiological divergences and compensatory effects in two *Leymus chinensis* (Poaceae) ecotypes in Northeast China.. Agriculture, Ecosystems & Environment.

[9] Wang RZ, Gao Q (2004). Morphological responses of *Leymus chinensis* (Poaceae) to the large-scale climatic gradient along the North-east China Transect (NECT).. Diversity and Distributions.

[10] Cellier F, Conejero G, Casse F (2000). Dehydrin transcript fluctuations during a day/night cycle in drought-stressed sunflower.. Journal of Experimental Botany.

[11] Peña-Rojas K, Aranda X, Joffre R, Fleck I (2005). Leaf morphology, photochemistry and water status changes in resprouting *Quercus ilex* during drought.. Functional Plant Biology.

[12] Jackson R, Sperry J, Dawson T (2000). Root water uptake and transport: using physiological processes in global predictions.. Trends in Plant Science.

[13] Sobrado M (2007). Relationship of water transport to anatomical features in the mangrove *Laguncularia racemosa* grown under contrasting salinities.. New Phytologist.

[14] Saradhi P, Aliaarora S, Prasad K (1995). Proline accumulates in plants exposed to UV radiation and protects them against UV-induced peroxidation.. Biochemical and Biophysical Research Communications.

[15] Wang RZ, Chen L, Bai YG, Xiao CW (2008). Seasonal Dynamics in Resource Partitioning to Growth and Storage in Response to Drought in a Perennial Rhizomatous Grass, *Leymus chinensis*.. Journal of Plant Growth Regulation.

[16] Li JD (1978). *Aneurolepidium chinense* grassland in China.. Journal of Northeast Normal University.

[17] Wang RZ, Gao Q, Chen Q (2003). Effects of climatic change on biomass and biomass allocation in *Leymus chinensis* (Poaceae) along the North-east China Transect (NECT).. Journal of Arid Environments.

[18] Zhang XS, Gao Q, Yang DA, Zhou GS, Ni J (1997). A gradient analysis and prediction on the Northeast China Transect (NECT) for global change study.. Acta Botanica Sinica.

[19] Chown S, Gaston K, Robinson D (2004). Macrophysiology: large scale patterns in physiological traits and their ecological implications.. Functional Ecology.

[20] Ehleringer J, Cooper T (1992). On the role of orientation in reducing photoinhibitory damage in photosynthetic-twig desert shrubs.. Plant, Cell & Environment.

[21] Maroco J, Pereira J, Manuela Chaves M (2000). Growth, photosynthesis and water-use efficiency of two C_4_ Sahelian grasses subjected to water deficits.. Journal of Arid Environments.

[22] Sack L, Cowan P, Jaikumar N, Holbrook N (2003). The ‘hydrology’of leaves: coordination of structure and function in temperate woody species.. Plant, Cell & Environment.

[23] Sack L, Holbrook N (2006). Leaf hydraulics.. Annual Review of Plant Biology.

[24] Sisó S, Camarero J, Gil-Pelegrín E (2001). Relationship between hydraulic resistance and leaf morphology in broadleaf *Quercus* species: a new interpretation of leaf lobation.. Trees-Structure and Function.

[25] Lu JM, Zhang CZ, Zhang HQ (1994). The character of morphology anatomy of monocotyledons in saline-alkali soil of resistant and the study physiological adaptability interrelation.. Journal of Northest Normal University.

[26] Pääkkönen E, Vahala J, Pohjola M, Holopainen T, Kärenlampi L (1998). Physiological, stomatal and ultrastructural ozone responses in birch (*Betula pendula* Roth.) are modified by water stress.. Plant, Cell & Environment.

[27] Calkin H, Gibson A, Nobel P (1986). Biophysical model of xylem conductance in tracheids of the fern *Pteris vittata*.. Journal of Experimental Botany.

[28] Gullo M, Salleo S, Piaceri E, Rosso R (1995). Relations between vulnerability to xylem embolism and xylem conduit dimensions in young trees of *Quercus corris*.. Plant, Cell & Environment.

[29] Bandurska H (2000). Does proline accumulated in leaves of water deficit stressed barley plants confine cell membrane injury? I. Free proline accumulation and membrane injury index in drought and osmotically stressed plants.. Acta Physiologiae Plantarum.

[30] Chandra Babu R, Shashidhar HE, Lilley JM, Thanh ND, Ray JD (2001). Variation in root penetration ability, osmotic adjustment and dehydration tolerance among accessions of rice adapted to rainfed lowland and upland ecosystems.. Plant Breeding.

[31] Hare P, Cress W (1997). Metabolic implications of stress-induced proline accumulation in plants.. Plant Growth Regulation.

[32] Munns R, James R, Läuchli A (2006). Approaches to increasing the salt tolerance of wheat and other cereals.. Journal of Experimental Botany.

[33] Ni J, Zhang X (2000). Climate variability, ecological gradient and the Northeast China Transect (NECT).. Journal of Arid Environments.

[34] Bates L, Waldren R, Teare I (1973). Rapid determination of free proline for water-stress studies.. Plant and Soil.

[35] Ebell LF (1965). Variation in total soluble sugars of conifer tissues with method of analysis.. Phytochemistry.

